# Cerebrospinal Drain Infection by *Candida auris*: A Case Report and Review of the Literature

**DOI:** 10.3390/jof10120859

**Published:** 2024-12-11

**Authors:** Asimenia Halioti, Charikleia S. Vrettou, Eleftherios Neromyliotis, Evdokia Gavrielatou, Aikaterini Sarri, Zoi Psaroudaki, Eleni E. Magira

**Affiliations:** 1 First Department of Critical Care Medicine, Evangelismos Hospital, Medical School, National and Kapodistrian University of Athens, 106 76 Athens, Greece; minaxal@yahoo.gr (A.H.); vrettou@hotmail.com (C.S.V.);; 2 Department of Neurosurgery, Evangelismos Hospital, National and Kapodistrian University of Athens, 106 76 Athens, Greece; 3 Department of Clinical Microbiology, “Evangelismos” General Hospital of Athens, 106 76 Athens, Greece

**Keywords:** *Candida auris*, cerebrospinal fluid, external ventricular drain, ventriculitis

## Abstract

*Candida auris* is notorious for its ability to spread within healthcare environments, particularly in intensive care units (ICUs), posing significant challenges for clinicians as treatment options become limited. This is especially concerning in the context of central nervous system (CNS)-invasive infections. While rare, its involvement in nosocomial brain ventriculitis presents substantial diagnostic and therapeutic challenges, with no established guidelines for managing CNS infections caused by *Candida auris*. This report presents a case of *Candida auris* ventriculitis in an ICU patient and offers a comprehensive and targeted literature review, emphasizing diagnostic approaches, treatment strategies, and the clinical complexities of managing this emerging pathogen in CNS infections.

## 1. Introduction

*Candida* species are among the leading fungal pathogens implicated in hospital-acquired infections (HAIs), particularly affecting traditionally immunocompromised patients, as well as individuals with prolonged hospital stays or those undergoing invasive medical procedures [1,2]. These infections, collectively referred to as candidiasis, range from superficial mucosal conditions to severe, life-threatening diseases such as bloodstream infections (candidemia) [3]. Candidemia, in particular, is associated with high morbidity and mortality rates in healthcare settings. A key factor contributing to the role of *Candida* in HAIs is its ability to form biofilms on medical devices, including central venous catheters, urinary catheters, and ventilators. These biofilms enable persistent colonization, making infections difficult to eradicate and significantly increasing resistance to antifungal treatments [4].

While *Candida albicans* has traditionally been the most frequently isolated species in clinical settings, recent years have seen an increase in infections caused by non-*albicans Candida* species, such as *Candida glabrata*, *Candida parapsilosis*, and the multi-drug-resistant *Candida auris* (*C. auris*) [5,6]. Since *Candida* is a highly heterogeneous genus of yeasts, the evolving field of taxonomy increasingly aligns species names with genetic and phylogenetic data. Therefore, the reclassification of species like *Candida glabrata* (now *Nakaseomyces glabratus*) and *C. auris* (proposed as *Candidozyma auris*) reflects significant advancements in our understanding of these organisms’ evolutionary relationships. *C. auris* has emerged as a particularly concerning pathogen due to its ability to survive on surfaces for prolonged periods, facilitating nosocomial transmission [5,6]. Most *C. auris* isolates are fluconazole-resistant and have a propensity to develop resistance to additional antifungal agents during treatment, although antifungal susceptibility profiles vary by clade. Over the past decade, outbreaks of *C. auris* have been reported in multiple countries, with a notable rise in cases during the COVID-19 pandemic [7].

Effective management of *Candida* infections in healthcare settings requires rigorous infection control measures. These include strict adherence to hygiene protocols, the use of rapid diagnostic techniques, and the timely initiation of appropriate antifungal therapy. Such measures are essential for limiting the spread of resistant strains and reducing the risk of adverse outcomes in affected patients [8,9,10]. The combination of intrinsic resistance to most available antifungal medications, challenges in identification using conventional techniques, lack of well-established preventive measures, thermoresistance, and partial resistance to commonly used disinfectants has led to *C. auris* being classified as a critical priority fungal pathogen by the World Health Organization (WHO) [11].

*C. auris* primarily affects critically ill and immunocompromised patients, causing both superficial and invasive infections, such as bloodstream infections, urinary tract infections, and central nervous system (CNS) infections [12,13]. Severely ill patients with neurological injuries and cerebrospinal fluid (CSF) drains, such as external ventricular drains (EVDs), are particularly at risk for developing intensive care unit (ICU)-acquired infections, including ventriculitis. Recent studies on the pathophysiology of ventriculitis suggest two primary mechanisms for pathogen invasion: retrograde movement of pathogens from the distal portion of the catheter lumen to the ventricular system or the direct introduction of pathogens during drain insertion [14,15,16]. Although ICU-acquired brain ventriculitis is relatively rare, it carries a substantial burden of morbidity and mortality, especially among critically ill patients [17,18,19,20].

The medical literature on hospital-acquired CNS infections caused by *C. auris* is limited [21,22,23,24,25,26,27,28,29,30,31] (Table 1). Managing these infections poses significant challenges due to diagnostic and therapeutic uncertainties [32]. Ideally, prompt removal of the infected CNS device should be performed [30], combined with the initiation of empirical intravenous antifungal therapy. However, the manipulation of EVDs to administer antimicrobial agents increases the risk of serious neurological complications [16,33]. Empirical and definitive treatment decisions must be guided by a thorough assessment of the available options, antifungal susceptibility testing, and the pharmacokinetic profiles of potential agents.

In response to the identification of *C. auris* in the cerebrospinal fluid of a patient in our department, we integrated a literature review into the case discussion to address this clinical challenge. We identified published cases of CNS- and EVD-related infections caused by this emerging pathogen and analyzed the clinical characteristics, diagnostic approaches, and management strategies reported in these cases. By doing so, we hope to familiarize clinicians with this poorly understood clinical entity and equip them to make informed decisions in similar cases.

## 2. Case Presentation

The patient, a man in his 50s, had been hospitalized in the neurosurgery department for nearly 10 days before being admitted to the ICU postoperatively following emergency external ventricular drain (EVD) placement for hydrocephalus and intraventricular hemorrhage. He had been diagnosed with chronic adult hydrocephalus five years earlier at another institution, but his past medical history was otherwise unremarkable. He experienced progressive clinical deterioration, characterized by behavioral changes and gait ataxia, which necessitated two valve replacements within one month. Over the following days, the EVD system required multiple manipulations due to dysfunction and blockage. During routine ICU screening, the patient tested positive for *C. auris* colonization. Although routine screening for *C. auris* in hospitals is not common globally, it is being increasingly implemented in areas with a higher prevalence of the pathogen or where previous outbreaks have occurred. We have adopted this practice due to prior outbreak history in our ICU. The screening methods we used involved swabbing patients’ groin, axilla, wounds, or areas around invasive devices (e.g., central lines, tracheostomies) with separate swabs. Although he exhibited no symptoms of active infection, and in anticipation of permanent ventriculoperitoneal (VP) shunting, a cerebrospinal fluid (CSF) examination on day 17 post-ICU admission revealed fungal spores. Diagnosing *C. auris* can be challenging because of its phenotypic similarity to other *Candida* species. VITEK 2 systems often fail to reliably identify *C. auris* and can lead to misidentification. Given the limitations of traditional culture methods, molecular techniques such as PCR, MALDI-TOF mass spectrometry, and DNA sequencing (e.g., ITS sequencing) are increasingly being used to accurately identify *C. auris*. In our case, MALDI-TOF was particularly helpful in identifying the pathogen with high accuracy. However, some contemporary systems may still misidentify *C. auris* as other *Candida* species, highlighting the importance of updating the databases used in such diagnostics. The accurate diagnosis of *C. auris* and reliable antimicrobial susceptibility testing are both crucial for effective treatment and infection control. Table 2 summarizes the laboratory findings for the patient treated in our ICU.

**Figure 1:**
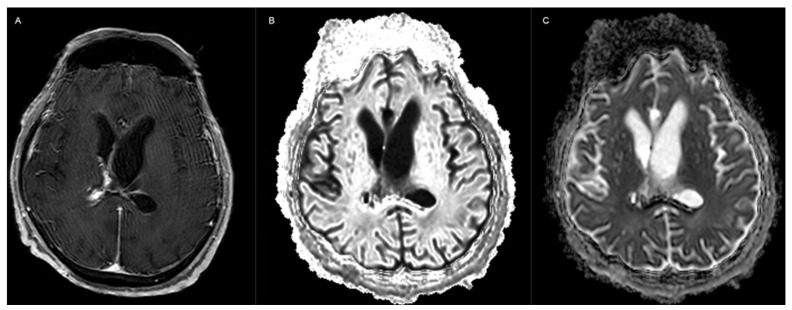
Magnetic resonance imaging scan (**A**) of the patient revealed contrast enhancement of the walls of the enlarged ventricular system (**B**) with restricted diffusion, consistent with ventriculitis (**C**).

The EVD was replaced, and fresh CSF samples confirmed the diagnosis of *C. auris* ventriculitis via CSF culture. Empirical antifungal therapy with liposomal amphotericin B was initiated while awaiting the antifungal susceptibility results. An MRI of the brain (Figure 1) showed contrast enhancement of the ventricular walls and restricted diffusion, findings which are consistent with ventriculitis. 

Given the seriousness of the infection, and after the antifungal susceptibilities were reported (Table 3), the antifungal regimen was escalated to include a combination of intravenous liposomal amphotericin B at 7 mg/kg/day and intravenous micafungin at 200 mg/day for a prolonged six-week course. Follow-up CSF cultures taken one week after starting antifungal therapy were negative for *C. auris*, demonstrating the efficacy of the treatment regimen. The patient’s condition steadily improved, and following the completion of antifungal therapy, a permanent VP shunt was successfully inserted. The patient was subsequently discharged from the ICU in a stable condition. Follow-up assessments at six weeks confirmed the continued absence of fungal infection.

## 3. Literature Review

This work was initiated in response to a clinically significant case of *C. auris* ventriculitis diagnosed in our department, which prompted an extensive review of the literature to gather current evidence on the subject. A systematic approach was undertaken to identify, compile, and analyze all existing reports of *C. auris*-related central nervous system (CNS) infections.

A Boolean search strategy was employed using the keywords “*Candida auris*” AND/OR “meningitis” AND/OR “ventriculitis” AND/OR “cerebrospinal fluid” AND/OR “external ventricular drain”. The search was conducted on the PubMed/MEDLINE and Scopus databases, chosen for their comprehensive coverage of the biomedical literature. No restrictions on publication year were applied to ensure all relevant evidence was included until the end of July 2024. Studies were eligible for inclusion if they reported confirmed cases of *C. auris* CNS infections with sufficient clinical details regarding diagnosis, treatment, and outcomes. Studies were excluded if they focused on other *Candida* species or lacked adequate diagnostic confirmation. 

Three independent reviewers (C.S.V., A.H., E.E.M.) screened the titles and abstracts of the identified records, followed by a detailed full-text review of potentially eligible articles. Any discrepancies in study selection were resolved through consensus. Data extraction focused on patient demographics, clinical presentation, diagnostic methods, therapeutic interventions, and outcomes. References from selected case reports were also manually reviewed to identify additional relevant studies. No publication year limit was applied to maximize the scope of the evidence reviewed.

**Figure 2:**
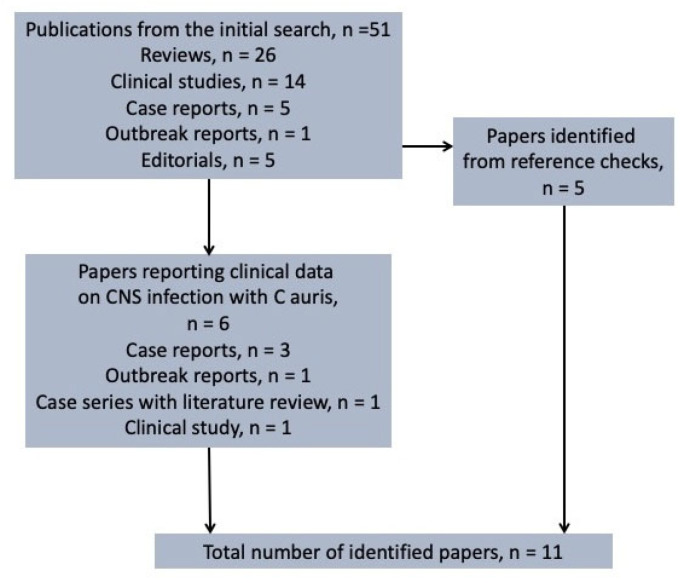
Search strategy for the identification of meningitis and ventriculitis cases, caused by *Candida auris,* until the end of July 2024. CNS: central nervous system.

Our search results are summarized in Figure 2. A total of 51 publications were identified, the majority of which were literature reviews. Among these, six publications specifically addressed *C. auris* CNS infections. These included three case reports [23,24,31], one outbreak report [26], one case series with a literature review [28], and one clinical study [25]. Additionally, by reviewing the references of the initially identified studies, we found several more reported cases of *C. auris* ventriculitis in the literature [21,22,27,29,30]. The published cases of ventriculitis and meningitis that were ultimately identified are presented in Table 1. These findings highlight that, despite the increasing recognition of *C. auris* as a significant healthcare-associated pathogen, specific reports involving CNS infections remain scarce in the literature.

## 4. Discussion

This case highlights the importance of the timely detection and appropriate management of *C. auris* infection, particularly in high-risk populations such as patients with prolonged hospitalizations, invasive devices, and those who have undergone surgical interventions. Hospital-acquired infections related to EVDs pose a significant risk to neurosurgical patients, particularly those in ICUs. EVDs are commonly used to manage conditions like hydrocephalus and intracranial hemorrhage by draining excess CSF [34]. However, their invasive nature increases the risk of infection, particularly ventriculitis, which involves inflammation of the brain’s ventricular system [35,36,37]. These infections can occur when pathogens, including bacteria and fungi, enter the sterile ventricular system either during drain placement or through subsequent manipulation of the device [38,39]. 

The risk of infection is heightened by factors such as prolonged EVD placement, frequent handling or adjustments of the drain, and the patient’s immunocompromised state [40,41]. Ventricular drain-associated infections are particularly dangerous for neurosurgical patients, as they can result in severe neurological complications, including increased intracranial pressure, brain abscesses, and even death [42,43]. These infections carry high morbidity and mortality, especially when not promptly identified and treated. Management requires preventive strategies such as strict adherence to aseptic techniques, timely removal of EVDs when no longer needed, and the initiation of empirical antimicrobial therapy if infection is suspected [44,45].

Reported cases of *C. auris* ventriculitis in the literature remain scarce, with most involving ICU patients [21,22,23,24,25,26,28,29,30,31]. Ventriculitis itself is relatively uncommon, with approximately 10% of patients with EVDs developing the condition, a figure that varies depending on the diagnostic criteria [36,46]. Advances in EVD management, standardized protocols, and improved handling have likely contributed to the limited number of reported cases [47,48]. Moreover, *C. auris* is a much rarer pathogen compared to more common causes of ventriculitis, such as Gram-positive organisms historically and Gram-negative bacteria (e.g., *Acinetobacter*) in recent years [47,48,49]. Underdiagnosis may also play a role, particularly in cases where *Candida* species coexist with other pathogens [48,50]. Despite its rarity, *C. auris* is significant, particularly following the COVID-19 pandemic, which has been linked to its emergence, especially in immunocompromised patients [50,51].

Patients at increased risk of candidiasis include neutropenic, immunosuppressed individuals, as well as non-neutropenic patients with comorbidities such as diabetes, AIDS, or extensive wounds (e.g., burns, surgical sites). Other high-risk groups include premature infants and patients requiring parenteral feeding or vascular catheters [52]. *C. auris* is notable for its persistence in hospital environments and on patients’ skin for extended periods. In our case, the patient was colonized with *C. auris* while on mechanical ventilation, a known risk factor for both colonization and invasive infection [20,53,54]. The implementation of screening protocols in high-prevalence areas and the use of effective treatment regimens play crucial roles in managing *C. auris* infections and preventing outbreaks in healthcare settings. Preventive measures and prompt treatment are therefore critical in susceptible patients [55,56].

Preventive strategies for *C. auris* colonization include patient screening using nasal, axillary, and groin swabs, with evidence supporting additional sites such as the rectum, pharynx, urine, wounds, and catheter exit points. Transmission-based precautions—such as single-room isolation, use of personal protective equipment, and disinfection of surfaces with agents like hydrogen peroxide or chlorine-based products—are essential. However, no specific intervention is currently proven to eliminate *C. auris* colonization [57].

Compared to bacterial ventriculitis, *C. auris* ventriculitis in critically ill patients tends to present more indolently. A pre-existing neurological condition combined with comorbidities may obscure the clinical assessment. Brain imaging, alongside CSF analysis and culture, is essential for diagnosis. While pyogenic ventriculitis is associated with fever and elevated inflammatory markers, these findings may be absent in *C. auris* infections [14,58,59,60]. In our case, contrast enhancement of the ventricular walls and periventricular edema on MRI suggested either infectious ventriculitis or persistent hydrocephalus. Timely CSF screening, particularly before permanent ventriculoperitoneal (VP) shunt insertion, is crucial in ICU patients with EVDs.

Clinicians face significant challenges in accurately diagnosing and treating *C. auris* CNS infections. The laboratory identification of *C. auris* is complex due to its phenotypic similarity to other species, such as *Candida haemulonii*, *Clavispora lusitaniae* (formerly *Candida lusitaniae*), and *Candida albicans* [59,60,61]. Advanced methods like matrix-assisted laser desorption/ionization time-of-flight (MALDI-TOF) mass spectrometry offer timely and accurate species identification, as was the case in our patient. However, such technology may not be readily available in low-resource settings [62].

Treatment options depend on antifungal susceptibility data. While no established susceptibility breakpoints exist for *C. auris*, the Centers for Disease Control and Prevention (CDC) has suggested tentative breakpoints based on expert opinion and those for closely related *Candida* species. These guidelines informed the selection of antifungal therapy in our case [63,64].

*C. auris* exhibits high minimum inhibitory concentrations (MICs) for various antifungal agents, with widespread resistance to fluconazole. Many isolates also demonstrate high MICs for newer azoles, such as voriconazole, though clinical breakpoints for these agents have not yet been established. While resistance to echinocandins remains uncommon, it is increasingly identified due to the widespread use of these drugs as first-line therapy, and pan-resistant strains have also been reported [62,65,66,67,68,69,70,71]. Variability in the MIC results for amphotericin B (AMB), depending on the antifungal susceptibility testing (AFST) method, can lead to the overestimation of amphotericin B resistance in *C. auris* [72]. Resistance to *amphotericin B* was over-reported with the commercial colorimetric method Sensititre YeastOne (SYO). SYO produced MICs that were one- to twofold dilutions higher than those of the reference CLSI method, resulting in 89% major errors. Major errors were reduced using a SYO-specific colorimetric wild-type upper limit MIC value of 8 mg/L (0%) or a 50% growth inhibition endpoint (3%) [72]. Therefore, amphotericin B MIC of the presented *C. auris* case at 2 mg/L was interpreted as susceptible based on the above. 

In our case, antifungal susceptibility testing (Table 3) expanded the options for designing an effective antifungal regimen. Knowing that the pathogen was sensitive to liposomal amphotericin B allowed us to include this antifungal in the treatment plan and avoid intrathecal echinocandin administration via the EVD. Unlike echinocandins, liposomal amphotericin B achieves adequate CNS and CSF penetration [73], making it a suitable option for treating brain ventriculitis. Due to the persistent hydrocephalus requiring multiple EVD manipulations and replacements, as well as the patient’s prior *C. auris* colonization and the necessity for permanent VP shunt placement, we opted for a combination of high-dose liposomal amphotericin B and high-dose micafungin [73]. This regimen was well tolerated, successfully cleared the CNS infection, and facilitated the subsequent insertion of the permanent VP shunt.

Compared to previously reported cases in the literature, this case highlights several important aspects of *C. auris* ventriculitis. Notably, the infection had an indolent presentation, complicating timely diagnosis and intervention. The patient’s prolonged EVD use and multiple manipulations were significant risk factors compounded by prior *C. auris* colonization. The imaging findings in this case were nonspecific, emphasizing the challenge of diagnosing ventriculitis based solely on radiological data. However, thorough laboratory testing, including advanced microbiological techniques, confirmed the diagnosis and guided the treatment strategy. Achieving therapeutic CSF concentrations of antifungal agents was critical, along with ensuring an appropriate duration and dosing regimen.

The presented case, along with the integrated literature review, has several limitations that warrant consideration. First, the scarcity of reported cases of *C. auris* ventriculitis limits the generalizability of the conclusions. The limited available data may not fully capture the spectrum of clinical presentations or outcomes associated with this rare condition. Additionally, much of the evidence stems from case reports or small case series, which lack the statistical power and rigorous controls of larger cohort studies or randomized controlled trials. This reliance on individual cases restricts the ability to draw robust conclusions about optimal diagnostic and treatment approaches. Variability in microbiological identification techniques across studies may also lead to the misidentification of *C. auris* or underreporting of its incidence, particularly in resource-limited settings where advanced diagnostic tools like MALDI-TOF are unavailable. Such variability could affect the recognition and classification of *C. auris* CNS infections.

The absence of standardized antifungal susceptibility testing and breakpoint guidelines specifically for *C. auris* CNS infections adds another challenge. Tentative breakpoints based on expert opinions and data from related *Candida* species remain provisional. Consequently, treatment regimens vary widely among reported cases, making it difficult to establish best practices or compare the efficacy of antifungal agents. The potential for the rapid development of antifungal resistance during treatment further complicates management. Additionally, the lack of long-term follow-up data in many cases limits assessments of treatment durability and recurrence risk.

Finally, this review focuses primarily on CNS infections and ventriculitis related to EVDs in ICU patients, potentially overlooking other manifestations of *C. auris* CNS involvement or infections in different patient populations. This narrower focus on EVD-related infections may bias conclusions toward interventions specific to this patient group, limiting broader applicability.

*C. auris* ventriculitis is a rare entity, and its clinical significance warrants vigilance among intensivists and healthcare providers managing patients with CNS foreign bodies. Prior *C. auris* colonization may increase the risk of infection. Accurate and timely diagnosis is essential, and antifungal susceptibility patterns must guide therapy to ensure optimal treatment, particularly before considering intrathecal administration. Prolonged courses of antifungal therapy and combination regimens may be required. Ultimately, neurological outcomes are largely influenced by the patient’s pre-existing condition and underlying pathology.

## Figures and Tables

**Table 1:** Reported cases of *Candida auris* ventriculitis and meningitis: clinical and laboratory characteristics.

Author	Clinical Presentation	Past Medical History/Comorbidities	CSF Findings	Other Laboratory Findings	Imaging	Antifungal Susceptibility	Antifungal Treatment	Outcome
Morales-López et al., 2017 [21]	NR	NR	NR	NR	NR	S: E	NR	NR
Chawla et al., 2018 [22]	Altered sensorium, prior EVD in situ	Tuberculous meningitis	Yeast cells, high PMCs	NR	NR	S:E, LAmB	E	Death
Singhal et al., 2018 [23]	Altered sensorium, meningitis, VP shunt in situ, pyrexia	IHD	Yeast cells, 445 cells, (76%PMNs), low Glu	NR	NR	S:E, F, V,	C + F/itC + C + F+ V/itC + V/V, 6 w	Survival
Khatamzas et al., 2019 [24]	aSAH, EVD, VP shunt	Diabetes, hypothyroidism	16 cells, (100% LCs)	NR	NS	NR	M, 62 d	Survival
Khatamzas et al., 2019 [24]	aSAH, EVD, pyrexia	None	20 cells, (100% LCs)	NR	NS	NR	LAmB, 14 days	Survival
Khatamzas et al., 2019 [24]	VP shunt, immunosuppression, CSF leak	NHL	36 cells, (83% LCs)	NR	NS	NR	LAmB+ F/M, 42 d	Survival
Sayeed et al., 2019 [25]	NR	NR	NR	NR	NR	NR	NR	NR
Garcia et al., 2020 [26]	NR	NR	NR	NR	NR	NR	NR	NR
Chandramati et al., 2020 * [27]	Respiratory failure, feed intolerance	NR	NR	NR	NR	NR	V	Death
Mirhendi et al., 2021 ** [28]	Meningitis, pneumonia, hydrocephalus	Necrotizing pneumonia, lobectomy	Yeast cells, 52 cells, (90% PMNs), low Glu	NR	NS	S: LAmB	Fluc/LAmB+ oral F	Survival
Stover et al., 2021 [29]	EVD-related meningitis	Hypertension, ICH	53 cells, (75% PMNs), low Glu	Increased WCC	NS	S:E, LAmB, F	LAmB + F	Death
Serrato et al., 2021 [30]	ICH, pyrexia	VP shunt	NR	Increased CRP and PC	NR	NR	LAmB + F	Survival
Sridharan et al., 2024 [31]	Headache and vomiting	Multiple procedures, VP shunt	Turbid csf, no cells	NR	MRI #	S:E, LAmB	LAmB + it LAmB, 28 d	Survival

* neonate, ** child, EVD: external ventricular drain, WCC: white cell count, PMCs: polymorphonuclear cells, LCs: lymphocytes, Glu: glucose, aSAH: acute subarachnoid hemorrhage, VP shunt: ventriculoperitoneal shunt, ICH: intracranial hemorrhage, CRP: C-reactive protein, PC: procalcitonin, CSF: cerebrospinal fluid, MRI: magnetic resonance imaging, it: intrathecal, NR: not reported, NS: nonspecific findings. E: echinocandins, C: caspofungin, M: micafungin, LAmB: liposomal amphotericin B, V: voriconazole, F: flucytosine, # ependymitis/leptomeningitis/obstructive hydrocephalus.

**Table 2:** Cerebrospinal fluid analysis and culture findings of a *Candida auris* EVD-related ventriculitis case.

CSF	Day of *Candida auris* Diagnosis	One Week Later	Six Weeks Later
Cells (number)	38	17	16
Neutrophils (number)	20	3	6
Lymphocytes (number)	18	14	10
Red cells (number)	650	39	274
Gram-stain report	Fungal spores	No microorganisms	No microorganisms
Culture report	*Candida auris*	No growth	No growth
Glucose (mg/dL)	62	59	68
Protein (mg/dL)	82	75	64
Lactate dehydrogenase (IU/L)	25	35	20

CSF, cerebrospinal fluid; EVD, external ventricular drain.

**Table 3:** Antibiotic susceptibility testing report of a case of *Candida auris* EVD-related ventriculitis.

Antifungal	MIC (mg/L)	Susceptibility Testing Report/ATU *
Anidulafungin	0.12	S
Micafungin	0.12	S
Caspofungin	0.25	S
Isavuconazole	0.12	N/A
Posaconazole	0.5	N/A
Voriconazole	1	N/A
Itraconazole	0.5	N/A
Fluconazole	≥256	R
Amphotericin B	2	S

* S: sensitive, R: resistant, N/A: non-applicable, ATU: area of technical uncertainty.
